# Mass drug administration for malaria elimination: do we understand the settings well enough?

**DOI:** 10.1186/s12916-018-1230-4

**Published:** 2018-12-19

**Authors:** Manuel W. Hetzel, Blaise Genton

**Affiliations:** 1 Swiss Tropical and Public Health Institute, P.O. Box, 4002 Basel, Switzerland; 2 University of Basel, Petersplatz 1, 4003 Basel, Switzerland; 3 Division of Infectious Diseases and Department of Community Health, University Hospital (CHUV), Rue du Bugnon 44, 1011 Lausanne, Switzerland

**Keywords:** Mass drug administration, Malaria, Malaria elimination, Low transmission, Ongoing transmission, Effectiveness

## Abstract

Mass drug administration (MDA) of antimalarials has re-emerged as a recommended tool for interrupting malaria transmission, but evidence from low endemicity settings is scarce. A trial in Zanzibar found that two rounds of MDA made no significant impact on malaria incidence, and many questions on the optimal mode and setting for implementing MDA remain unanswered. A more thorough understanding of local sources and drivers of transmission, and a better toolbox for evaluating interventions in near-elimination settings are essential.

Please see related research article: https://bmcmedicine.biomedcentral.com/articles/10.1186/s12916-018-1202-8.

## Background

As the World Health Organization reports stagnation in the fight against malaria [1], urgent efforts are needed to avoid the reversal of recent gains. In some places, this may mean increasing the coverage of vector control, such as long-lasting insecticidal nets (LLIN) and indoor residual spraying, or improving access to efficacious treatments, including for cases of *Plasmodium vivax* or artemisinin-resistant *P. falciparum.* Other places may have exhausted the available repertoire of standard tools, and complementary interventions are required to tackle ongoing transmission. In pre-elimination settings, low level transmission appears to be particularly difficult to tackle because interventions must be increasingly targeted, requiring a more thorough understanding of local patterns of transmission and a robust and adaptable health system [2].

Mass drug administration (MDA), the time-limited administration of antimalarial treatment to everyone at about the same time and at repeated intervals [1], has re-emerged as a recommended tool to accelerate the interruption of transmission in areas approaching elimination [3]. Easily applicable diagnostic tools are simply not yet sensitive enough to detect the last parasite; hence they leave large parts of the asymptomatic reservoir undetected [4]. Interestingly, the recommendation for MDA is based on weak evidence, particularly with regard to demonstrating impact in low endemic African settings [5], though modelling studies provide some theoretical guidance [6].

In a trial conducted in Zanzibar by Morris and colleagues [7], two rounds of MDA with dihydroartemisinin-piperaquine (DHAP) were used, plus a single low dose of primaquine (SLD PQ). The well-designed cluster-randomised trial found no impact on malaria incidence and only a marginal and transient effect on the prevalence of asymptomatic infections, as determined by polymerase chain reaction (PCR). These results are sobering, especially given that the setting appeared operationally conducive and a reasonable coverage level with DHAP was achieved, for which modelling suggested a notable impact [6]. Furthermore, while coverage with SLD PQ could have been higher, it is uncertain whether much better operational coverage could be achieved under routine programme conditions.

The authors attribute the lack of impact to suboptimal timing (during rather than before the peak malaria season), a possibly insufficient number of MDA rounds, and the importance of importation of infections. In the context of the latter, it is misleading to refer to their outcome as “transmission” because this is not what was assessed. Measuring transmission in near-elimination settings remains a unique challenge; therefore – as in the study by Morris et al. – proxy measures are applied [8]. However, in Zanzibar and similar settings, the relative contributions of local transmission and imported infections remain an important factor of uncertainty, despite several previous investigations, and the reliability of routinely reported travel history is unclear [9].

The measurement of adherence using blood concentration of piperaquine in intervention areas (as was done in the study by Morris et al.) is welcome because it is known that self-reporting of medication intake is unreliable [10, 11]. However, it would have been useful to measure the blood concentration of a variety of antimalarials in both intervention and control areas. Widespread unreported use of antimalarials (obtained from health facilities and private retailers) has been documented in Tanzania [11] and might have modified the effect of MDA rounds.

The results from this study are nevertheless highly relevant. Firstly, they underline the importance and value of real-world data generated by well designed epidemiological and intervention studies to validate the prediction of models. Secondly, as transmission declines and heterogeneity increases, a thorough understanding of the target of malaria control efforts is required. This is particularly true in environments with a high transmission potential. In the study by Morris et al., prevalence by PCR differed considerably between intervention and control areas. It increased in intervention areas (from 0.8% to 1.7%) and decreased in control areas (from 2.5% to 1.4%), suggesting that the study did not capture important drivers of the local epidemiology. Thirdly, indicators for measuring the effect of interventions in low transmission settings must be carefully chosen. Small changes are difficult to detect, minor contextual fluctuations (and major ones, such as the redistribution of LLIN) may mask intervention effects, and sufficient detail is required to explain unexpected study outcomes. In such a context, it is particularly important – albeit difficult – to identify the source of local infections because not all may represent local transmission.

## Conclusions

Many questions about the optimal mode and setting for implementing MDA remain unanswered. However, trials, such as the one by Morris et al., make a useful contribution to the evidence base on how to address ongoing transmission in near-elimination settings. Ideally, they would be complemented by more in-depth investigations of the drivers of local malaria epidemiology.
